# Report of Eleven Patients of Subcutaneous Panniculitis-Like T-Cell Lymphoma: Clinicopathologic Features, ^18^F-FDG PET/CT Findings and Outcome

**DOI:** 10.3389/fonc.2021.650822

**Published:** 2021-07-01

**Authors:** Maoqing Jiang, Long Zhao, Jianjun Zheng, Jingfeng Zhang, Ping Chen, Wenlan Zhou

**Affiliations:** ^1^ Ningbo PET/CT Center, Hwa Mei Hospital, University of Chinese Academy of Sciences, Ningbo, China; ^2^ Ningbo Institute of Life and Health Industry, University of Chinese Academy of Sciences, Ningbo, China; ^3^ Nanfang PET Center, Nanfang Hospital, Southern Medical University, Guangzhou, China; ^4^ Department of Nuclear Medicine, Xiamen Cancer Center, The First Affiliated Hospital of Xiamen University, Xiamen, China; ^5^ Department of Nephrology, Hwa Mei Hospital, University of Chinese Academy of Sciences, Ningbo, China

**Keywords:** subcutaneous panniculitis-like T-cell lymphoma, positron emission tomography/computed tomography, ^18^F-FDG avid, prognosis, clinicopathologic features

## Abstract

**Objectives:**

Subcutaneous panniculitis-like T-cell lymphoma (SPTCL) is a fairly rare subtype of primary cutaneous lymphoma. This study aims to investigate the clinicopathologic features, ^18^F-FDG PET/CT findings, and outcome of patients with SPTCL.

**Methods:**

A retrospective single-center study enrolled 11 patients with SPTCL between August 2010 and March 2020. A total of 26 ^18^F-FDG PET/CT scans were performed, and the initial and follow-up PET/CT imaging features, clinicopathologic and immunohistochemical characteristics, and outcome were analyzed.

**Results:**

The male-to-female ratio was 1.2. The mean age at diagnosis was 24.2 years (age range: 13–48 years). Histopathological examinations revealed atypical T-lymphocyte rimming of individual subcutaneous adipocytes, mostly with CD2^+^, CD3^+^, CD4^−^, CD5^+^, CD8^+^, CD56^−^, T-cell intracellular antigen-1^+^, Granzyme B^+^, and high Ki-67 index. Multiple large skin ulcerations with a maximum diameter of 10 cm were observed in one of the 11 patients (9.1%, 1/11), and hemophagocytic syndrome was found in another one. At initial PET/CT scans, the lesions in all 11 patients showed increased uptake of ^18^F-FDG with a wide range of maximum standard uptake value (SUVmax) from 2.0 to 14.9. The morphology of the lesions presented as multiple nodules and/or disseminated plaques mainly involving the trunk and/or limbs. Five patients had extracutaneous non-lymph node lesions with SUVmax of 5.6 ± 2.8 on ^18^F-FDG PET/CT. No significant correlation between SUVmax and Ki-67 index was observed (r = 0.19, *P* > 0.05). Follow-up ^18^F-FDG PET/CT scans in six patients showed complete remission of the disease in two, partial remission in three, and progressive disease in one. During the follow-up period, there was no death except for the patient with multiple ulcerations who died 4 months after diagnosis of SPTCL.

**Conclusions:**

SPTCL may be a group of heterogeneous diseases with varying degrees of ^18^F-FDG uptake. ^18^F-FDG PET/CT demonstrates its usefulness in detecting disease extent, providing diagnostic work-up, staging, and evaluating treatment response of SPTCL. Multiple large skin ulcerations may be a factor of poor prognosis for patients with SPTCL.

## Introduction

Subcutaneous panniculitis-like T-cell lymphoma (SPTCL) is a fairly rare subtype of primary cutaneous T-cell lymphoma, accounting for <1% of all cutaneous lymphomas (1). It was first reported by Gonzalez et al. in 1991 as a new subtype of T-cell lymphoma, which was always mimicking panniculitis and often complicated by hemophagocytic syndrome (HPS) (2). Initially, two subtypes, *α*/*β* and *γ*/*δ* T-cell phenotypes, were contained in SPTCL (3). The former generally has a favorable outcome, while the latter has a poor prognosis (4). In 2005, the term SPTCL was restricted to *α*/*β* T-cell phenotype by the World Health Organization European Organization for Research and Treatment of Cancer (EORTC) classification (5). In the recent update of WHO-EORTC classification, SPTCL and cutaneous *γ*/*δ* T-cell lymphoma were two distinct entities (6).

The involvement of subcutaneous adipose tissue is the main clinical feature of SPTCL, mostly presenting as multiple nodules and/or diffuse plagues (7). The histopathological features of SPTCL are numerous neoplastic T-lymphocytes rimming individual adipocytes and positive for CD8 and CD3, but negative for CD4 on immunohistochemistry according to EORTC classification (5, 8). However, the molecular phenotypes of SPTCL are still based on a few case studies and need to be further assessed. Besides, no typical imaging features were found on computed tomography (CT), ultrasound, and magnetic resonance imaging (MRI) (9, 10). Moreover, the extracutaneous non-lymph node disease of SPTCL, *e.g.* involvement of central nervous system, mesentery or intra-abdominal visceral fat, usually presented as negative on morphological imaging modalities (11–13). The imaging features of ^18^F-fluorodeoxyglucose (FDG) positron emission tomography/computed tomography (PET/CT) have been reported previously (11, 14–17). However, they are confined to case reports or reviews (18). The role and characteristics of ^18^F-FDG PET/CT in SPTCL have not yet been well established.

Although the clinical, histopathological, and immunohistochemical features have been illustrated before, the correlations between ^18^F-FDG PET/CT findings, clinicopathologic features, and outcome have not been well confirmed. Therefore, in this study, the aims were (i) to investigate the clinicopathologic features, ^18^F-FDG PET/CT findings, and outcome for patients with SPTCL; (ii) to explore the correlation between ^18^F-FDG PET/CT findings and immunohistochemical phenotypes; (iii) to find out the role of ^18^F-FDG PET/CT in staging and evaluating the treatment response for patients with SPTCL.

## Materials and Methods

### Patients

This retrospective study was approved by the institutional review board of Nanfang Hospital, Southern Medical University. Informed consent requirements were waived for the nature of this study. We performed a retrospective single-center study of 11 patients with SPTCL that was diagnosed by histopathology and immunohistochemistry between August 2010 and March 2020 in Nanfang Hospital (Southern Medical University, China). We carefully reviewed the clinicopathologic and immunohistochemical analyses regarding the patients using the WHO-EORTC classification for cutaneous lymphoma. The results of physical examinations, B-symptoms, complete blood count, lactate dehydrogenase (LDH), *β*_2_-microglobulin, ferritin, and bone marrow biopsy were obtained in all patients to assess the systemic involvement. The diagnosis of hemophagocytic syndrome (HPS) was based on the criteria of Hemophagocytic Lymphohistiocytosise-2004 (19).

All 11 patients underwent a total of 26 ^18^F-FDG PET/CT scans, and six of them had two or more PET/CT scans before and after treatment, and the remaining five only had an initial PET/CT scan. Out of the six patients, two had five PET/CT scans, one had four, one had three, and the remaining two had only two in the course of treatment response monitoring and follow up.

### Histopathologic and Immunohistochemical Analysis

Histological examinations of skin incision biopsy specimens were performed in the 11 patients. Tissue sections were stained with hematoxylin–eosin and immunohistochemical staining markers. Immunostaining was performed by antibodies against T-cell antigens (CD2, CD3, CD4, CD5, CD8, and CD56), B-cell antigens (CD20 and CD79a), cytotoxic proteins [granzyme B (GZB) and T-cell intracellular antigen-1 (TIA-1)] and the proliferation marker Ki-67. The results of Epstein–Barr virus (EBV) *in situ* hybridization were available in eight cases. All results were interpreted by reviewing medical records.

### ^18^F-FDG PET/CT Protocol

^18^F-FDG PET/CT scans were performed on a Biograph mCTx scanner (Siemens, Erlangen, Germany) in nine patients, and the remaining two were scanned using a GE Discovery LS PET/CT scanner (GE Healthcare, Waukesha, WI, USA). All patients were instructed to fast for at least 6 h, and blood glucose level was monitored to ensure the level within normal limits (<7.0 mmol/L). Approximately 60 min after the intravenous injection of 144–467 MBq (3.9–12.6 mCi, 150 μCi/kg) of ^18^F-FDG, whole-body PET/CT scan was performed according to the guidelines for tumor imaging with PET/CT 1.0 (20). ^18^F-FDG with a radiochemical purity >95% was manufactured automatically using the tracer synthesis system of a Tracerlab FXF-N (GE Healthcare, Waukesha, WI, USA; Beijing PET Biotechnology Co., Ltd., China). The corresponding low-dose CT was acquired at 80 mA and 140 kV (GE Healthcare) or automatic mA (Siemens). The acquired CT and PET images were sent to a Xeleris or MMWP workstation for registration and fusion. Lesions with focally increased ^18^F-FDG uptake exceeding that of the surrounding normal tissues were considered positive lesions. The region of interest (ROI) was drawn along the margin of the lesion on the PET image, and the maximum standardized uptake value (SUVmax) was measured.

### Evaluation of PET/CT Images

Two experienced nuclear medicine physicians analyzed all PET/CT images comparatively by frame and reached a consensus (MJ and WZ). Positive PET results were defined when the uptake of ^18^F-FDG in the focal or diffuse lesions was higher than the background, which cannot be explained by physiological activity (21, 22). Lymph nodes were classified as abnormal if ^18^F-FDG uptake was increased and/or abnormal in size (diameter >10 mm) regardless of ^18^F-FDG uptake. If extra-nodal lesions showed focally increased uptake of ^18^F-FDG and/or pathological anatomic features on CT scan (regardless of ^18^F-FDG uptake), they were considered abnormal (21). Combined with pre- and post-treatment CT or PET/CT findings, disease remission, progression or relapse were determined using the international working group consensus response evaluation criteria in lymphoma (RECIL 2017) (23). SPTCL was staged according to the TNM classification system for primary cutaneous lymphomas other than mycosis fungoides and Sezary syndrome (24).

### Survival and Statistical Analysis

The overall survival (OS) was calculated as the time from diagnosis of SPTCL to the date of death or last follow-up. The variables were expressed as mean ± standard deviation. The statistical comparison of SUVmax in different groups was analyzed by unpaired Student t test. The results of the difference between the comparative tests were considered statistically significant at a two-tailed value of *P <*0.05. The correlation between SUVmax and Ki-67 index was tested using linear regression analysis. All statistical analyses were performed using GraphPad Prism version 5.01.

## Results

### Clinical Features and Laboratory Results

**Figure 1:**
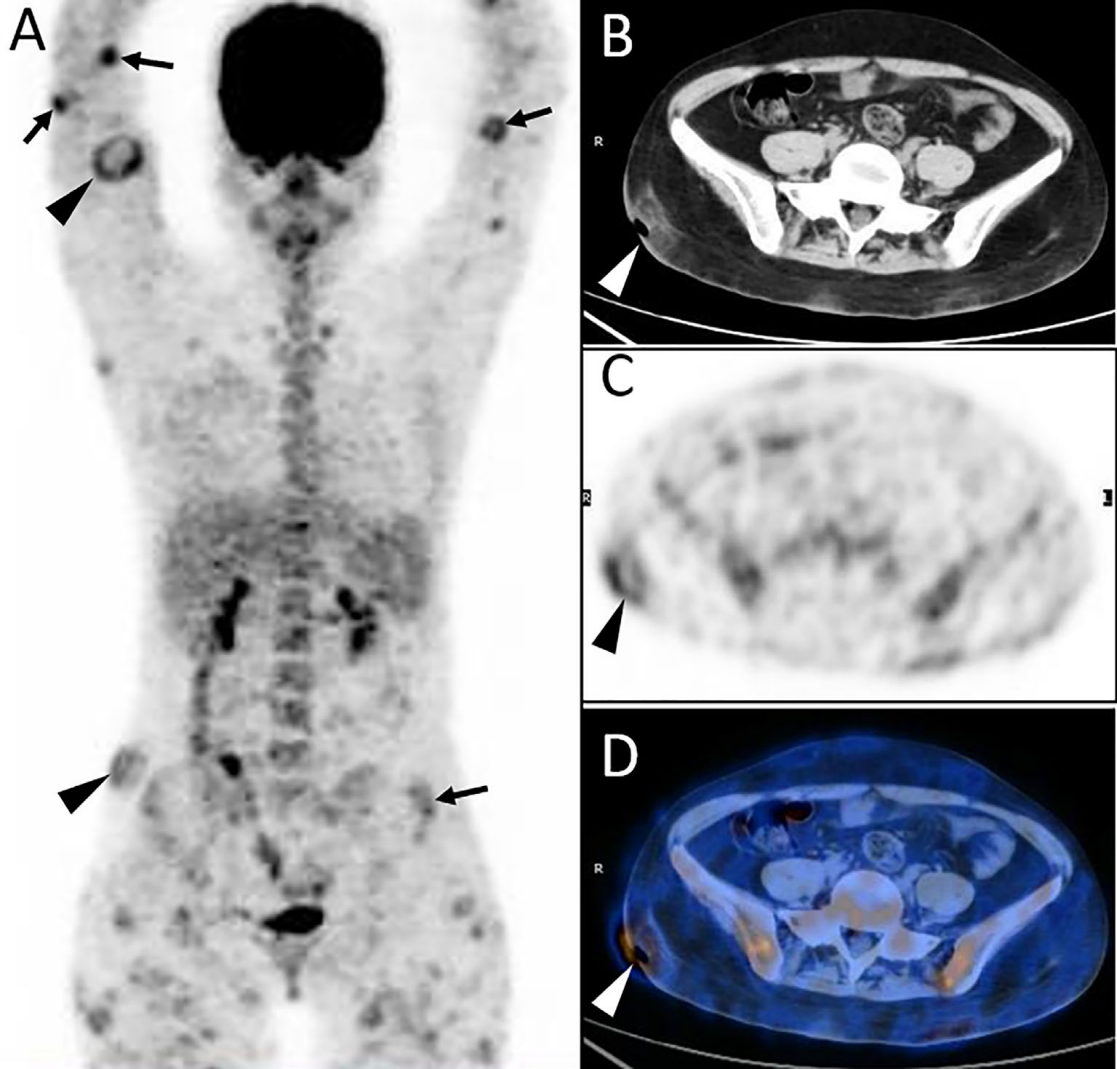
Twenty-year-old female with subcutaneous panniculitis-like T-cell lymphoma (SPTCL). Three-dimensional maximum intensity projection PET image **(A)** shows multiple nodules and diffuse plaques with high uptake of ^18^F-FDG in both upper arms and legs, lower back, and buttock. Multiple skin ulcerations with a maximum diameter of 10 cm present as circular uptake of ^18^F-FDG (arrowheads) on PET/CT scan. Abdominal axial CT **(B)**, corresponding PET **(C)**, and fusion PET/CT **(D)** images from ^18^F-FDG PET/CT clearly show the defection of skin in the right hip, which represents ulcer caused by lymphoma involvement. This patient died 4 months after diagnosis of SPTCL.

The clinical features and laboratory results of the 11 patients (six males and five females) are summarized in **Table 1**. The mean age at diagnosis was 24.2 years (age range: 13–48 years). The morphology of the lesions varied greatly from multiple nodules to disseminated plagues mainly involving in the subcutaneous adipose. The diameter of the localized lesions is 0.5 to 10 cm. The sites of subcutaneous nodules and/or diffuse plagues were predominantly on the trunk (9/11, 81.2%), upper (8/11, 72.7%), and lower limbs (7/11, 63.4%). Fever was the main clinical symptom occurring in nine of 11 patients (81.2%). More than half of the patients showed anemia (6/11, 54.5%), increased lactate dehydrogenase level (7/9, 77.8%), elevated *β*_2_-microglobulin level (7/8, 87.5%), and elevated ferritin (9/9, 100%). Multiple skin ulcerations (**Figure 1**) with a maximum diameter of 10 cm were observed in one patient, mainly involving both upper arms, right hip, and left upper leg. Only one patient had HPS complication, but this patient had a favorable prognosis after chemotherapy.

**Table 1:** Clinical features and laboratory results of the 11 patients with subcutaneous panniculitis-like T-cell lymphoma.

Characteristics	
Mean age at diagnosis, years (range)	24.2 (13–48)
Sex, n (%)	
Male	6/11 (54.5)
Female	5/11 (45.5)
Presenting skin lesions, n (%)	
Plaque	9/11 (81.2)
Nodule	4/11 (36.4)
Swelling	4/11 (36.4)
Ulcer	1/11 (9.1)
Extent of cutaneous involvement, n (%)	
Solitary	1/11 (9.1)
Localized	4/11 (36.4)
Multiple	9/11 (81.2)
Site, n (%)	
Face	2/11 (18.2)
Trunk	9/11 (81.2)
Upper extremities	8/11 (72.7)
Lower extremities	7/11 (63.4)
Extracutaneous lesions, n (%)	
Lymphadenopathy	3/11 (27.3)
Bone marrow involvement	2/11 (18.2)
Mesentery	3/11 (27.3)
Pleura	1/11 (9.1)
Abnormal laboratory test results, n (%)	
Leukopenia/lymphopenia	3/11 (27.3)
Thrombocytopenia	5/11 (45.5)
Anemia	6/11 (54.5)
Pancytopenia	2/11 (18.2)
Increased lactate dehydrogenase level	7/9 (77.8)
Elevated *β*2-microglobulin level	7/8 (87.5)
Elevated ferritin	9/9 (100)
Hemophagocytic syndrome, n (%)	1/11 (9.1)
B-symptoms, n (%)	
Fever	9/11 (81.2)
Weight loss	4/11 (36.4)
No fever and wight loss	1/11 (9.1)

### Histopathology and Immunophenotype

**Figure 2:**
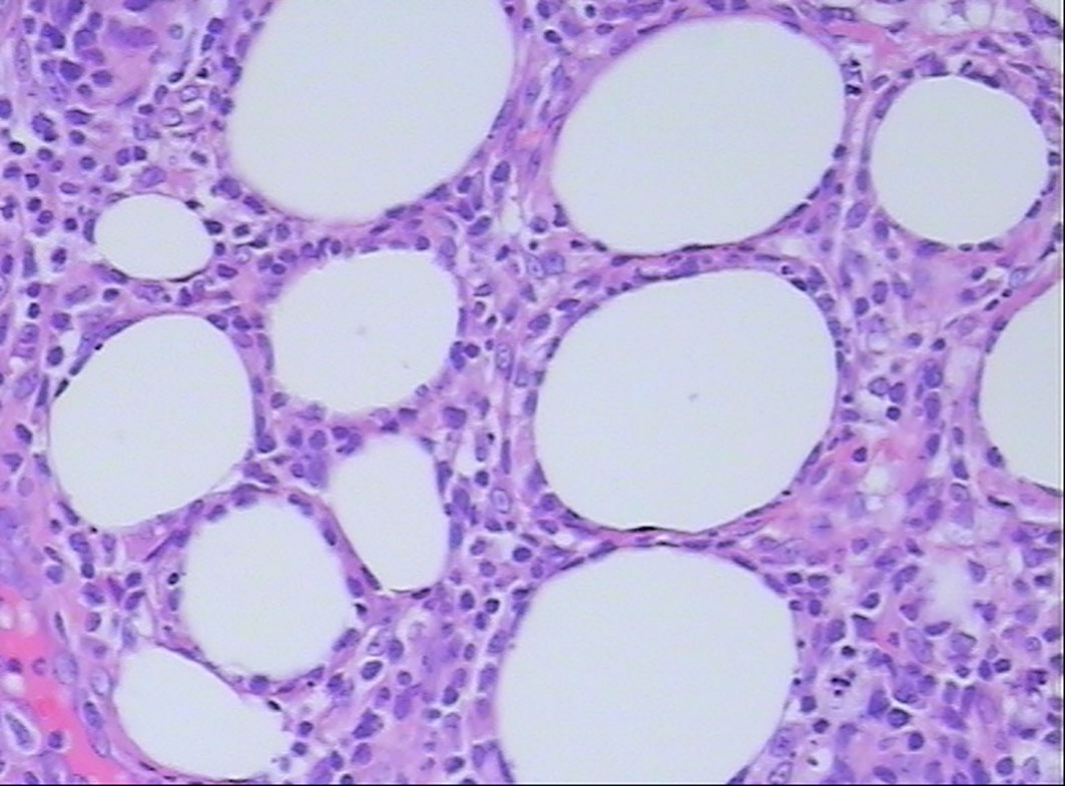
Histopathological examination shows atypical lymphocyte rimming of individual adipocytes (hematoxylin and eosin, 400×).

All patients were confirmed to be SPTCL by histopathological examinations, which showed numerous atypical neoplastic T-lymphocytes rimming individual fat cells (**Figure 2**). The epidermis and dermis were usually uninvolved unless associated with ulcerations (**Figure 1**). The immunophenotype results are summarized in **Table 2**. All cases were positive for CD2 (9/9), CD3 (11/11), CD5 (7/7), CD8 (10/10), TIA-1 (10/10), and GZB (9/9), and most of them were negative for CD56 (9/11). Four of 11 patients were positive for CD20, and five of six patients were positive for CD79a. All eight patients with available results were negative for EBER. In addition, the proliferation marker Ki-67 was positive in all cases, and approximately more than 70% of the patients (8/11) were higher than 50%.

**Table 2:** Immunohistochemical features of the 11 cases with subcutaneous panniculitis-like T-cell lymphoma.

Case No.	CD2	CD3	CD4	CD5	CD8	CD20	CD56	CD79a	TIA-1	GZB	Ki-67	EBER
1	NA	+	−	NA	+	+	+	+	+	+	+,60%	NA
2	+	+	−	+	+	−	−	−	+	+	+,90%	−
3	+	+	+	+	+	+	−	+	+	+	+,40%	−
4	+	+	+	NA	+	+	−	NA	+	+	+,50%	−
5	+	+	+	+	+	−	−	NA	+	+	+,80%	−
6	+	+	+	NA	+	+	−	+	+	+	+,70%	NA
7	+	+	−	+	+	−	−	NA	+	NA	+,18%	−
8	+	+	−	+	+	−	−	NA	+	+	+,60%	−
9	+	+	NA	+	NA	–	–	+	+	+	+,60%	−
10	NA	+	NA	NA	+	+	+	+	+	+	+,80%	NA
11	+	+	+	+	+	–	–	NA	NA	NA	+,90%	−0/8
Total	9/9	11/11	5/9	7/7	10/10	4/11	9/11	5/6	10/10	9/9	11/11	

NA, data not available; TIA-1, T-cell intracellular antigen-1; GZB, Granzyme B; EBER, Epstein–Barr virus-encoded small nuclear RNA.

### Initial and Follow-Up PET/CT Findings

**Figure 3:**
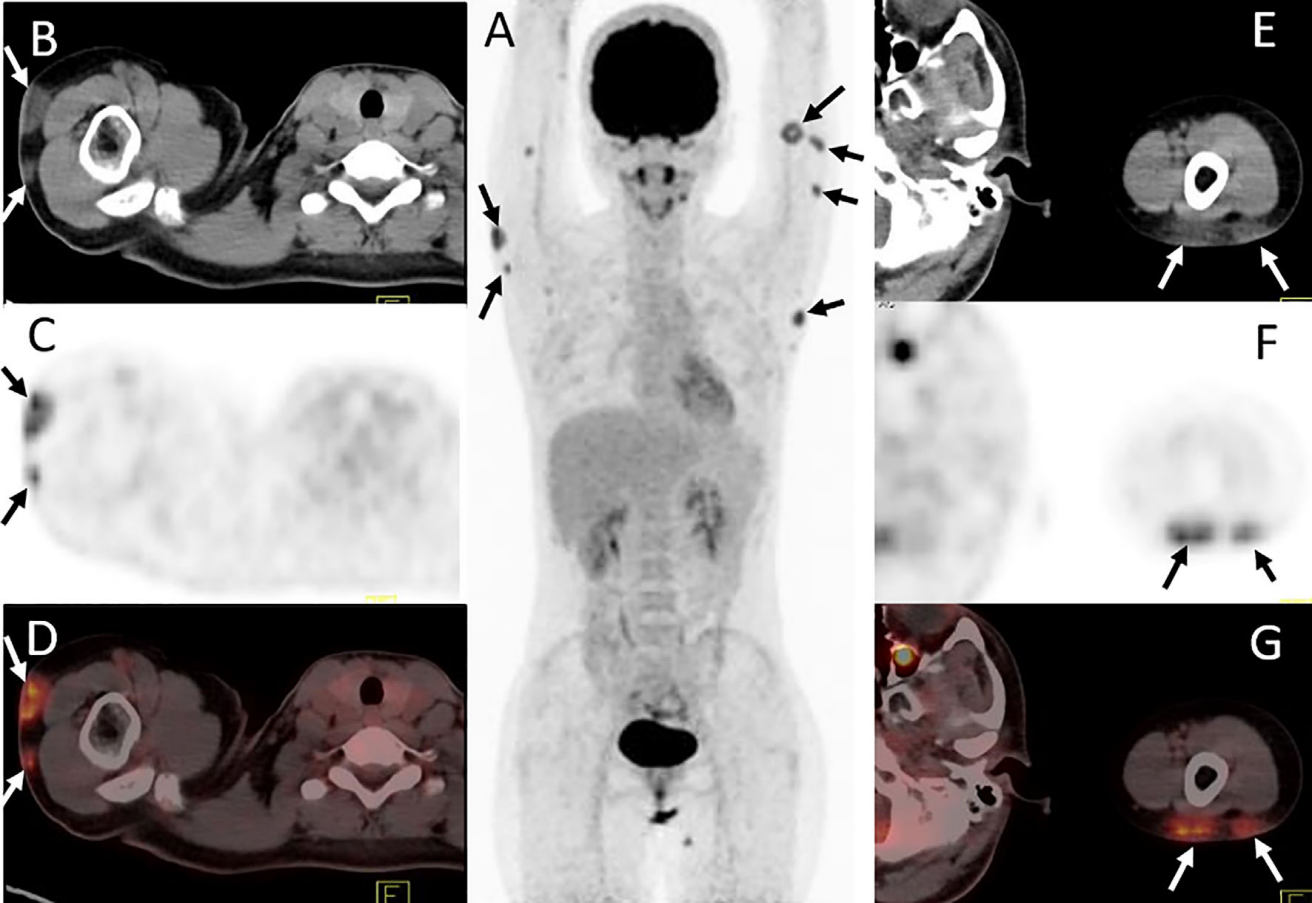
Twenty-four-year-old female with subcutaneous panniculitis-like T-cell lymphoma. Three-dimensional maximum intensity projection image **(A)**, axial CT **(B, E)**, corresponding PET **(C, F)** and fusion PET/CT **(D, G)** images from ^18^F-FDG PET/CT clearly show multiple intensely ^18^F-FDG-avid subcutaneous infiltrative nodules in both upper arms with poor or well defined borders (arrows), representing sites of lymphoma.

**Figure 4:**
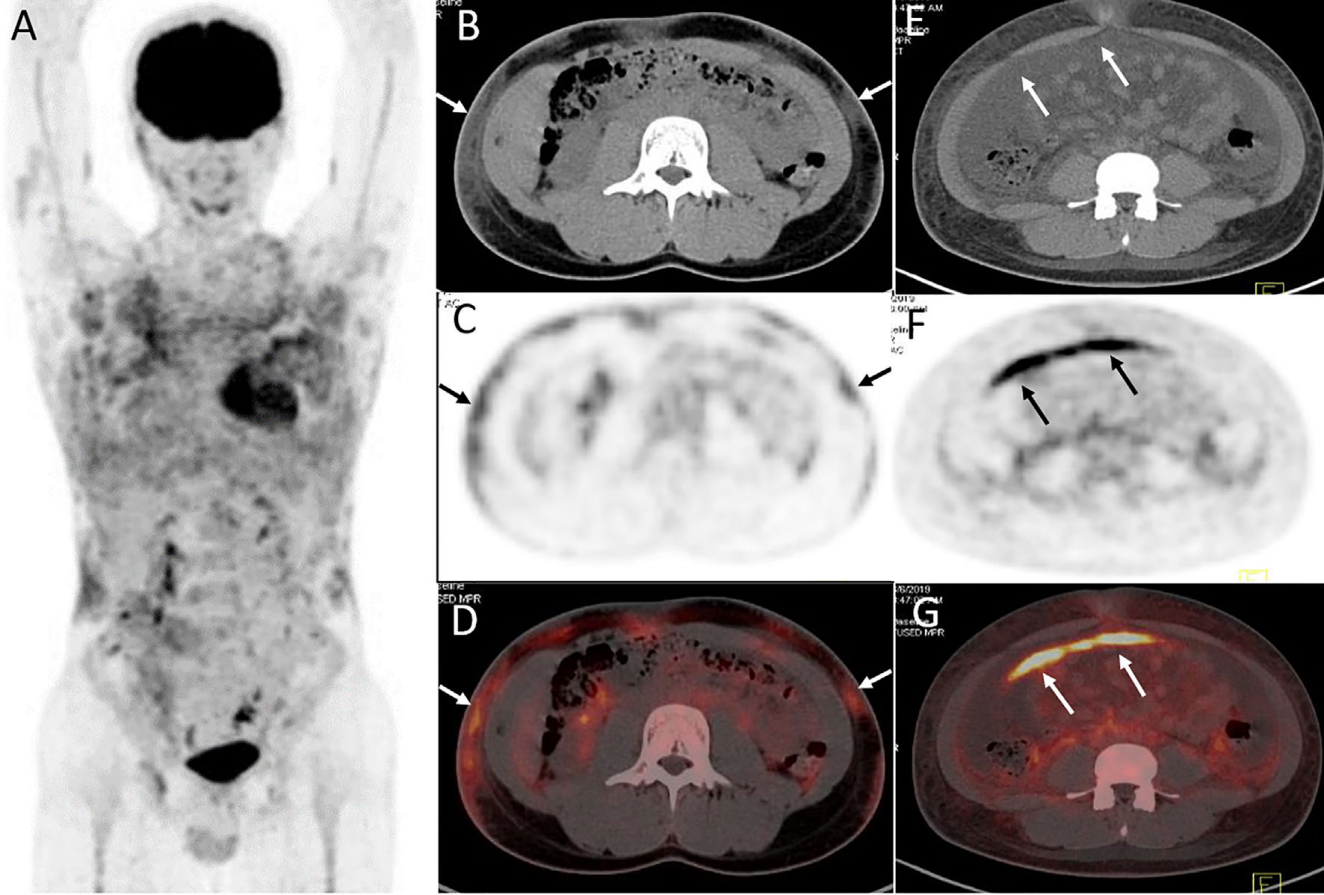
Twenty-year-old male with subcutaneous panniculitis-like T-cell lymphoma. Three-dimensional maximum intensity projection image **(A)**, axial CT **(B)**, corresponding PET **(C)** and fusion PET/CT **(D)** images from ^18^F-FDG PET/CT show hypermetabolic soft-tissue infiltrates subcutis diffusely in the trunk and both upper arms with ill-defined borders (arrows), which are the typical patterns of the disease. Abdominal axial CT **(E)**, corresponding PET **(F)**, and fusion PET/CT **(G)** images from ^18^F-FDG PET/CT clearly show a “strip-like” lesion with high uptake of ^18^F-FDG in mesentery, which represents lymphoma involvement but it is negative on CT scan.

At initial PET/CT scans, all lesions in the subcutaneous adipose showed increased uptake of ^18^F-FDG with a wide range of SUVmax from 2.0 to 14.9 (mean ± standard deviation, 5.0 ± 3.5). Mild and moderate uptake of ^18^F-FDG, which was lower than or similar to that of the liver, with SUVmax less than 3.5 was noted in five patients; while intense uptake of ^18^F-FDG, which was significantly higher than that in the liver, was found in the other six patients (**Table 3**). The morphology of the lesions varied greatly from multiple nodules (**Figure 3**) to disseminated or diffuse plaques (**Figure 4**), mainly involving the subcutaneous adipose of the trunk and/or upper or lower limbs.

**Table 3:** The simplified staging of subcutaneous panniculitis-like T-cell lymphoma by ^18^F-FDG PET/CT according to TNM classification system.

No.	Sex/age	SUVmax of subcutaneous lesions	TNM Staging	Skin or subcutaneous involvement			Lymph node involvement				Extracutaneous non–lymph node disease	
				Solitary	Regional	Generalized	0	1	≥2	Central	No evidence	Present
1	F/20	4.6	T3N0M0	No	No	Yes	Yes	No	No	No	Yes	No
2	M/25	3.0	T3N3M1	No	No	Yes	No	Yes	Yes	Yes	No	Yes
3	F/24	4.3	T2N0M0	No	Yes	No	Yes	No	No	No	Yes	No
4	M/48	3.4	T1N0M0	Yes	No	No	Yes	No	No	No	Yes	No
5	M/18	2.8	T3N0M0	No	No	Yes	Yes	No	No	No	Yes	No
6	F/15	14.9	T3N2M1	No	No	Yes	No	No	Yes	No	No	Yes
7	M/46	2.0	T3N0M0	No	No	Yes	Yes	No	No	No	Yes	No
8	M/14	4.3	T3N0M1	No	No	Yes	Yes	No	No	No	No	Yes
9	M/20	6.0	T3N0M1	No	No	Yes	Yes	No	No	No	No	Yes
10	F/13	3.1	T3N0M1	No	No	Yes	Yes	No	No	No	No	Yes
11	F/23	6.1	T3N2M0	No	No	Yes	No	No	Yes	No	Yes	No

**Figure 5:**
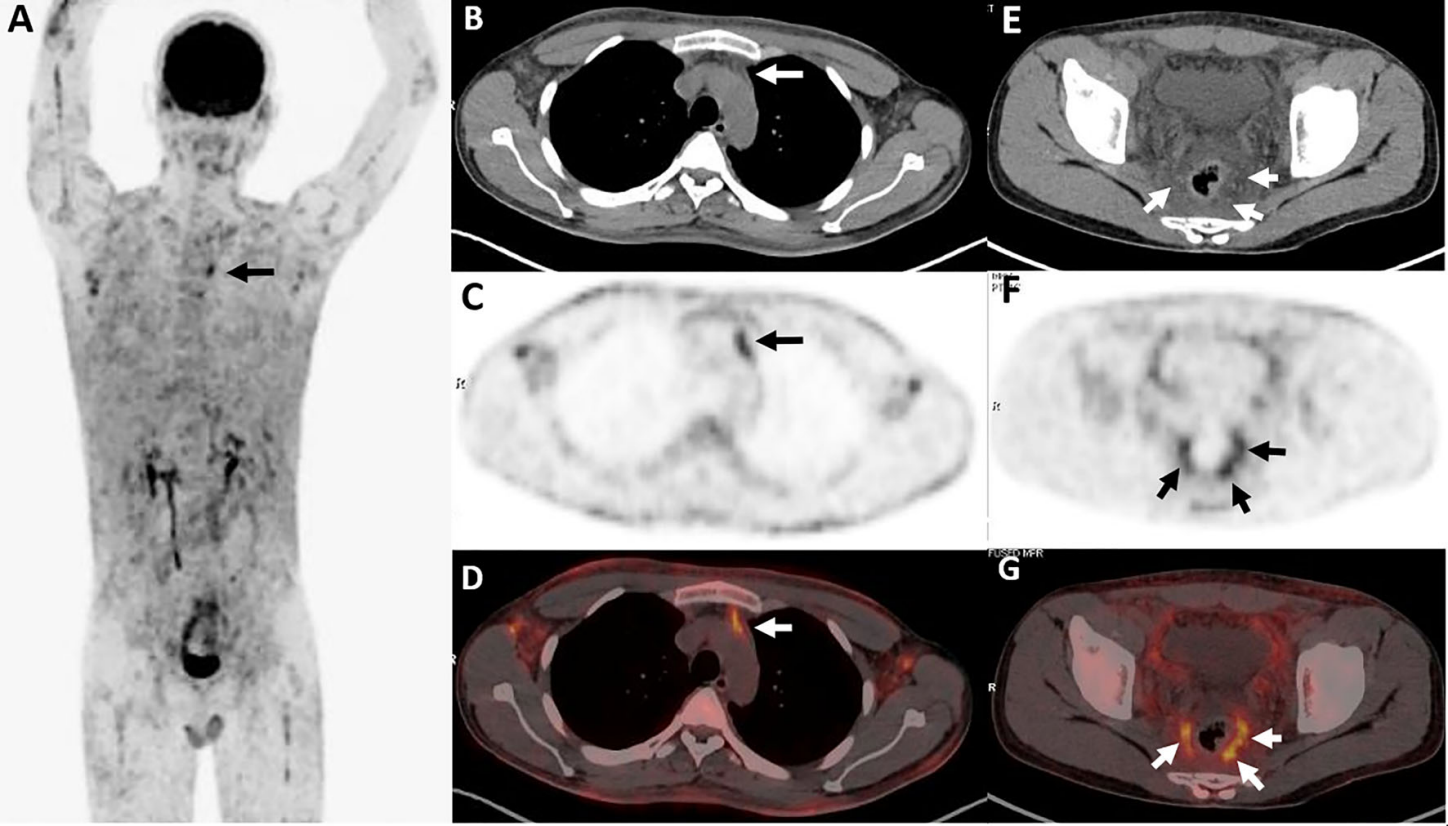
Twenty-five-year-old male with subcutaneous panniculitis-like T-cell lymphoma. Three-dimensional maximum intensity projection PET image **(A)** shows diffuse hypermetabolic lesions in the subcutaneous of the body. Thoracic axial CT **(B)**, corresponding PET **(C)** and fusion PET/CT **(D)** images show a poorly defined lesion with high uptake of ^18^F-FDG in the anterior mediastinum (arrows). Pelvic axial CT **(E)**, corresponding PET **(F)**, and fusion PET/CT **(G)** images show perirectal lesions are positive on PET scan but negative on CT scan.

Lesions were classified according to the TNM classification system; **Table 3** shows the staging of the 11 patients. Five patients had extra-cutaneous non-lymph node lesions, including mediastinum, paravertebral, perirenal, mesentery, peri-rectal, and right parotid gland. Most of them were negative on CT scans, whereas they were clearly observed on ^18^F-FDG PET/CT scans (**Figures 4E–G** and **5**) owing to the increased uptake of ^18^F-FDG (SUVmax, 5.6 ± 2.8). No significant difference of ^18^F-FDG uptake was revealed between subcutaneous and extra-cutaneous lesions (t = 0.42, *P* = 0.68).

**Figure 6:**
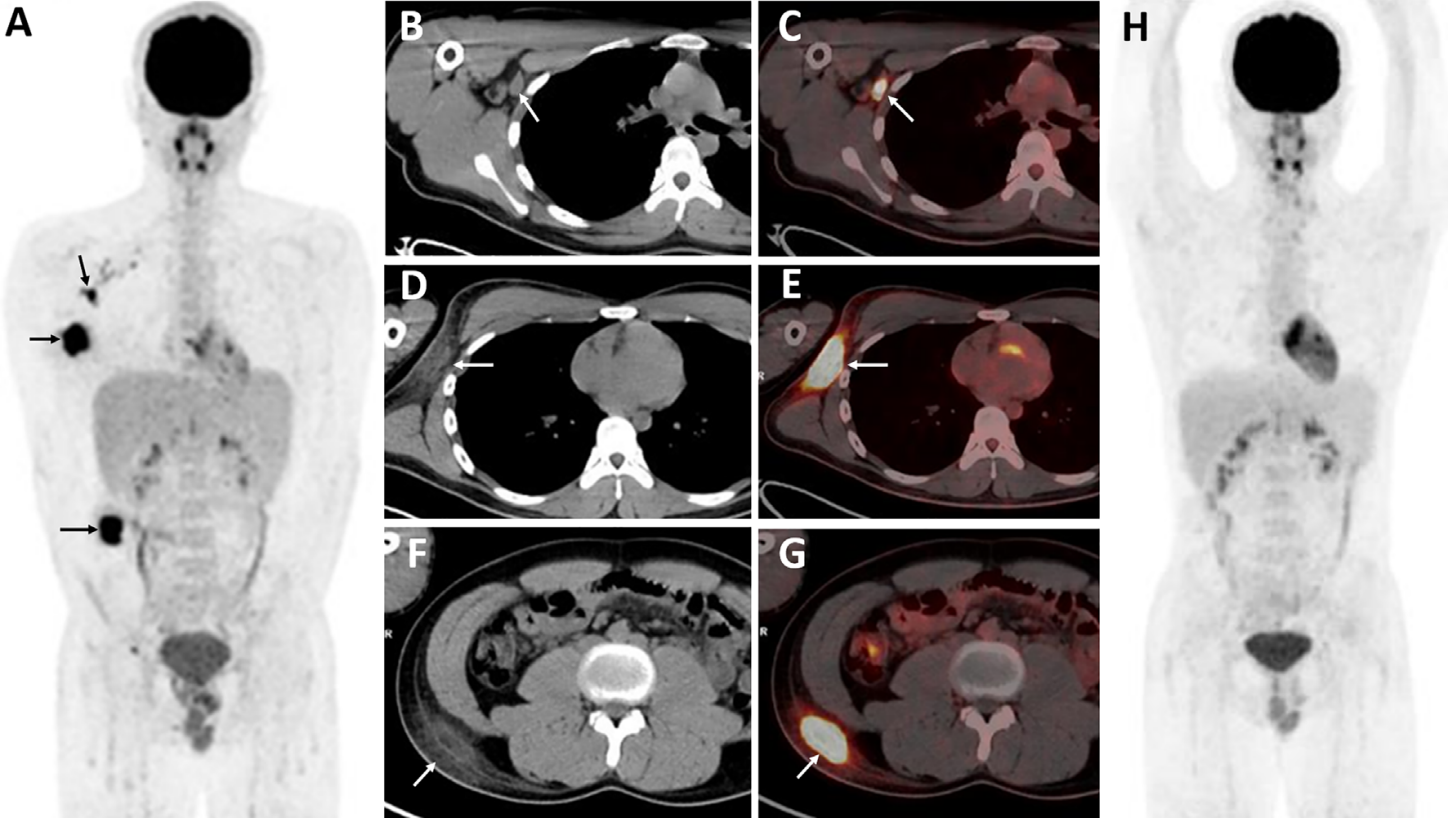
Eighteen-year-old male with subcutaneous panniculitis-like T-cell lymphoma. Three-dimensional maximum intensity projection image **(A)** shows three intensely ^18^F-FDG-avid lesions in the right side of the body (black arrows). Corresponding axial CT **(B, D, F)** and fusion PET/CT **(C, E, G)** images clearly show an enlarged lymph node with increased uptake of ^18^F-FDG in the right axillary and ill-defined lesions in the subcutaneous adipose of the body, representing sites of lymphoma. The patient achieved complete remission after chemotherpay **(H)**.

Enlarged lymph nodes with increased ^18^F-FDG uptake in the axillae and/or inguinal region were observed in three patients (**Figures 6B–C**). Six patients underwent follow-up ^18^F-FDG PET/CT scans, which revealed complete remission of the disease in two (**Figure 6**), partial remission in three, and progressive disease in one.

### Outcome and the Correlation Between SUVmax and Ki-67 Index

**Figure 7:**
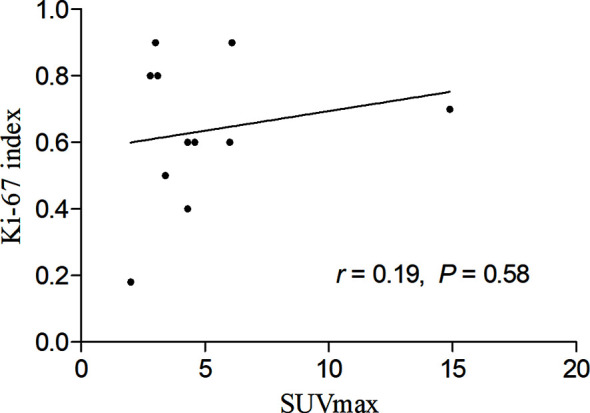
Linear regression analysis showing no significant correlation between maximum standardized uptake value (SUVmax) in the subcutaneous adipose lesions of the 11 patients with subcutaneous panniculitis-like T-cell lymphoma and proliferation index (Ki-67).

During the follow-up period, there was no death except for the patient with multiple skin ulcerations who died 4 months after diagnosis of SPTCL. The correlation between SUVmax of the lesions in the subcutaneous adipose on ^18^F-FDG PET/CT scan and the expression of Ki-67 of the lesions in the subcutaneous adipose on immunohistochemistry was calculated by linear regression analysis, which revealed no significant correlation between them in patients with SPTCL (r = 0.19, *P* > 0.05) (**Figure 7**).

## Discussion

In the present study, we investigated the clinicopathological features, ^18^F-FDG PET/CT findings, and outcome in 11 patients with SPTCL. Our results suggested that SPTCL might be a group of heterogeneous diseases characterized by multiple nodules and/or disseminated plagues mainly involving the subcutaneous adipose in the trunk and/or upper or lower limbs. The immunophenotypes revealed positive for CD2, CD3, CD5, CD8, TIA-1, and GZB, and negative for EBER in all tested patients. SPTCL was also a disease of ^18^F-FDG avid, but the intensity of ^18^F-FDG uptake varied greatly. The lesions were often confined in the subcutaneous adipose tissue, sometimes involving the extracutaneous non-lymph node tissues but rarely associated with lymph nodes and muscles. ^18^F-FDG PET/CT demonstrated to be useful in detecting disease extent, staging, and evaluating the treatment response of SPTCL. Moreover, we evaluated the correlation between SUVmax of the subcutaneous adipose lesions on ^18^F-FDG PET/CT scan and the proliferation marker Ki-67 of the 11 patients, but no significant correlation was obtained between the two parameters.

Clinically, patients with SPTCL often present as multiple subcutaneous nodules and/or plaques that are poorly circumscribed in the limbs and/or trunk, as well as exhibit an indolent clinical course and have a favorable prognosis (7, 25). The 5-year overall survival rate of SPTCL patients without complication of hemophagocytic syndrome (HPS) was greater than 90% (25). Although HPS had been reported as a factor of poor prognosis for patients with SPTCL (26), it was not associated with mortality in our results. Cutaneous *γ*/*δ* T-cell lymphoma often showed involvement of (epi)dermal and/or ulceration and poor prognosis (25). Even if it is rare in SPTCL, one of our 11 cases showed multiple large skin ulcerations in both upper arms, right hip, and left upper leg with a maximum diameter of 10 cm. The patient died 4 months after diagnosis of SPTCL, suggesting that large ulcerations might be a poor prognostic factor not only for cutaneous *γ*/*δ* T-cell lymphoma but also for STPCL. Of note, the majority of our patients showed high levels of lactate dehydrogenase (77.8%), *β*_2_-microglobulin (87.5%), and ferritin (100%), which might represent the laboratory characteristics of STPCL and help in establishing the diagnosis of SPTCL.

Histologically, SPTCL is usually characterized by atypical neoplastic T-lymphocytes that involve the subcutis with rimming of individual adipocytes. Epidermis and dermis are rarely involved unless with ulceration, which is preferentially in cutaneous *γ*/*δ* T-cell lymphoma. In our study, the immunohistochemical profile of SPTCL was predominately with CD2^+^, CD3^+^, CD8^+^, TIA-1^+^, GZB^+^, CD56^−^ and EBER^−^. The phenotype of CD4^+^/CD8^+^ is a rare subtype of SPTCL as reported previously (27), whereas it could be seen in five of nine cases in our study. No significant difference of clinicopathologic features was found when compared to the phenotype of CD4^−^/CD8^+^. Of particular note, Ki-67 staining was shown to be as high as 40–0% positive in cytotoxic CD8^+^ T-lymphocytes, which was similar to previous studies (27), confirming that Ki-67 staining is a highly specific factor for the diagnosis of SPTCL (28).

It is well known that ^18^F-FDG PET/CT plays a critical role in the diagnosis, staging, response evaluation, and prognosis prediction of ^18^F-FDG-avid lymphoma, such as diffuse large B-cell lymphoma, Hodgkin’s lymphoma, etc. (29, 30). However, due to its rarity, the role of ^18^F-FDG PET/CT in SPTCL has not been well established. The typical features of SPTCL on ^18^F-FDG PET/CT were multiple subcutaneous nodules or diffuse plagues with increased ^18^F-FDG accumulation (15, 18). From published reports (18), the imaging features of ^18^F-FDG PET/CT in SPTCL were only limited to subcutaneous lesions. Very few studies showed the findings of lymphadenopathy and extracutaneous involvement. Besides, the intensity of ^18^F-FDG uptake has not been well illustrated previously. Barrington et al. (30) showed the percentage of ^18^F-FDG avidity was 71% in SPTCL, whereas Feeney et al. (31) found that all SPTCL lesions were positive on ^18^F-FDG PET/CT scans. Our results were consistent with Feeney et al. that all lesions presented a high uptake of ^18^F-FDG but with varying degrees (mean SUVmax 5.0, range 2.0–14.9). In addition, the activity of ^18^F-FDG between subcutaneous and extracutaneous non-lymph node lesions revealed no significant difference, suggesting that glucose metabolism of these lesions is homologous. From ^18^F-FDG PET/CT scans, three patients revealed lymphadenopathy, and five patients showed extracutaneous non-lymph node involvement. But most of these lesions were negative on conventional CT scan. Therefore, our results demonstrate that ^18^F-FDG PET/CT is useful in providing the burden of tumor, detecting disease extent, and staging of SPTCL.

Moreover, the final diagnosis of SPTCL still depends on immunohistochemistry, whereas ^18^F-FDG PET/CT plays an important role in providing the diagnostic suggestion of SPTCL and guiding the biopsy. Patients with SPTCL often show high uptake of ^18^F-FDG on PET/CT, which will be valuable for identifying the biopsy site. Evaluating treatment response and detecting the occult extracutaneous involvement of SPTCL by ^18^F-FDG PET/CT had been demonstrated to be useful in previous studies (15, 32). Our results also showed that PET/CT might be helpful in detecting the subcutaneous and extracutaneous non-lymph node lesions, providing the biopsy site, staging, and monitoring treatment response for patients with SPTCL. The correlation of Ki-67 and SUVmax on PET/CT had been evaluated in non-Hodgkin lymphoma (NHL). Chang et al. (33) showed a significant correlation between the two parameters in newly diagnosed NHL. However, the Ki-67 index and SUVmax revealed no significant correlation in our 11 case series. The possible reasons may be the limited numbers and the different sites between the measurement of SUVmax and the biopsy site of Ki-67. Thus, further studies are needed to assess the correlation between the two parameters.

Our study had some limitations. Even though it is the largest case series to show ^18^F-FDG PET/CT findings and the role of PET/CT in patients with SPTCL, it is a retrospective study and the number of patients still is small due to rarity of this disease. In addition, the treatment strategies varied a lot, and it is difficult to provide a suitable plan for therapy. Last but certainty not the least, not all patients were followed up with ^18^F-FDG PET/CT; the follow-up time was not the same, and the number of scans was also different.

In summary, we demonstrate that patients with SPTCL have a favorable outcome in addition to complication of multiple large ulcerations and predominantly present as multiple subcutaneous adipose lesions with a wide range of morphology from multiple nodules to diffuse plagues. Both subcutaneous and extracutaneous lesions show high uptake of ^18^F-FDG with varying degrees. ^18^F-FDG PET/CT demonstrates to be valuable in detecting disease extent, providing diagnostic work-up, staging, and monitoring treatment response for patients with SPTCL. Moreover, no correlation between SUVmax and Ki-67 index was observed in our case series. Overall, more studies are required to validate these findings.

## Data Availability Statement

The raw data supporting the conclusions of this article will be made available by the authors, without undue reservation.

## Ethics Statement

This retrospective study was approved by the institutional review board of Nanfang Hospital, Southern Medical University. Informed consent requirements were waived for the nature of this study.

## Author Contributions

MJ and WZ conceived the idea of the study. MJ, LZ, JJZ, JFZ, PC, and WZ collected the data. MJ, LZ, and WZ performed image analysis. MJ wrote the manuscript. MJ, LZ, and WZ performed the statistical analysis. LZ, JJZ, JFZ, PC, and WZ edited and reviewed the manuscript. All authors contributed to the article and approved the submitted version.

## Funding

This work was supported by the Medical Scientific Research Foundation of Zhejiang Province, China (Grant no. 2021KY1014), Research Foundation of Hwa Mei Hospital, University of Chinese Academy of Sciences, China (Grant no. 2021HMKY05), Medical Science and Technology Project of Ningbo, China (Grant no. 2020Y10), and Medical Research Foundation of Guangdong province (Grant no. A2016590), and Guangdong Natural Science Foundation (Grant no. 2016ZC0070).

## Conflict of Interest

The authors declare that the research was conducted in the absence of any commercial or financial relationships that could be construed as a potential conflict of interest.
